# Dengue incidence and length of viremia by RT-PCR in a prospective observational community contact cluster study from 2005–2009 in Indonesia

**DOI:** 10.1371/journal.pntd.0011104

**Published:** 2023-02-06

**Authors:** Silvita Fitri Riswari, Dyana Safitri Velies, Nurhayati Lukman, Ungke Anton Jaya, Hofiya Djauhari, Chairin Nisa Ma’roef, Khin Saw Aye Myint, Susana Widjaja, Andre van der Ven, Bachti Alisjahbana, Quirijn de Mast, Herman Kosasih

**Affiliations:** 1 Health Research Unit, Faculty of Medicine, Faculty of Medicine, Universitas Padjadjaran, Bandung, Indonesia; 2 Department of Internal Medicine and the Radboud Center for Infectious Diseases, Radboud University Medical Center, Nijmegen, The Netherlands; 3 Viral Diseases Program, U.S. Naval Medical Research Unit No.2, Jakarta, Indonesia; 4 Eijkman Institute for Molecular Biology, Jakarta, Indonesia; 5 Department of Internal Medicine, Hasan Sadikin General Hospital, Bandung, Indonesia; University of Florida, UNITED STATES

## Abstract

**Background:**

Dengue has become a major global health threat since being recognized three centuries ago. Important gaps remain in understanding the transmission dynamics of dengue virus (DENV) infection. This study reports the results of a prospective observational cluster study that investigated the incidence of symptomatic and asymptomatic infections and length of viremia among close community contacts of hospitalized DENV-infected patients.

**Methodology/principal findings:**

Between 2005 and 2009, dengue-confirmed cases (n = 97) admitted to Hasan Sadikin Hospital in Bandung, Indonesia, were enrolled as index cases. Subsequently, twenty close community contacts (n = 1928) living with and around the index cases were included and followed up for up to 14 days. Body temperature was measured daily; blood samples were collected every 3–4 days and when reported fever. DENV infection was confirmed using Reverse Transcriptase–Polymerase Chain Reaction (RT-PCR), IgM rapid test, and Enzyme-linked Immunosorbent Assay (ELISA). Among the 1928 community contacts, a total of 72 (3.7%) acute DENV infections were diagnosed, which equates to an incidence of 636 cases per 1,000 person-years (95% Confidence interval (CI) 588 to 687 cases per 1,000 person-years). Twenty-nine cases (40%) were symptomatic (22 dengue fever (DF) & 7 dengue hemorrhagic fever (DHF)), and 43 (60%) were asymptomatic. Primary and secondary DENV infections were detected in 18 (25%) and 54 (75%) subjects. Among the RT-PCR positives, viremia was observed as early as seven days before fever onset and converted to negative as late as seven days after the onset of fever.

**Conclusions:**

DENV infections are common among close community contacts of hospitalized dengue patients. The high number of asymptomatic infections and the observation that viremia precedes the onset of fever for up to seven days highlight the importance of unrecognized dengue transmission and the need for improved transmission control.

## Author summary

Data regarding a complete course of dengue viremia, especially in pre-febrile and asymptomatic cases, are limited. These data can be obtained from community cluster studies that enroll and follow up with the neighborhood and household members of dengue-confirmed index cases. Using this approach, we assessed the presence of dengue viremia before the development of fever and the incidence of symptomatic and asymptomatic dengue cases within the community contacts. We found that there are 1.5 asymptomatic dengue infections for each symptomatic case. Furthermore, dengue virus in blood was detected in eight out of sixteen post-enrollment dengue subjects before the onset of fever. The maximum period was up to seven days, longer than previously reported. Viremic subjects play a crucial role in dengue transmission by mosquitoes. Our data indicate that the significance of unnoticed virus transmission supports the need for comprehensive and sustainable vector control interventions. With the active surveillance embedded in the study design, we calculated the incidence of dengue infection to be 636 cases per 1,000 person-years of follow-up, 930 times higher than the national incidence rate around the same study period, showing the real burden of disease in a hyperendemic area and confirming the underestimation of Indonesian incidence data.

## Introduction

Dengue is an important arboviral disease caused by four dengue virus (DENV) serotypes (DENV 1–4) which were first transmitted from human to human by the bite of the *Aedes aegypti* mosquito around three centuries ago [1]. The disease is endemic in more than 100 countries and has become an enormous threat to public health [1,2]. The incidence of DENV infection is commonly underestimated, especially in endemic countries. It is difficult to distinguish it from other causes of acute febrile illnesses due to the limited use of specific diagnostics. Registered dengue cases commonly come from symptomatic hospitalized cases, resulting in inaccurate estimates of disease burden and a lack of outbreak response in the endemic areas [3].

Important gaps remain in our understanding of the transmission dynamics of DENV infection, including the length of dengue viremia in both symptomatic and asymptomatic cases [4]. More importantly, information regarding the complete course of dengue viremia, especially in pre-febrile and asymptomatic cases, is limited [4,5].

In this report, we conducted a prospective dengue cohort using a cluster study design in Bandung, a highly populated city in West Java, Indonesia. We enrolled close community contacts from hospitalized dengue patients. These community contacts were followed prospectively to determine the incidence of symptomatic and asymptomatic infections and the onset of viremia that systematically analyses the development of asymptomatic and symptomatic cases [4,6–7].

## Methods

### Ethical clearance

This dengue community cluster study was approved by the institutional review boards of the National Institute of Health Research and Development, the Indonesian Ministry of Health no. LB.03.02/KE/4828/08. Written informed consent was obtained from all study participants. For children participants, we obtained written informed consent from their parents or guardians.

### Study population and design

This study was conducted in Bandung, West Java, from 2005 to 2009, as an expansion of the previous study carried out by our group in Jakarta [8]. The trial design is summarized in **S1 Fig.** To initiate a cluster; we enrolled dengue-confirmed cases by Reverse Transcriptase–Polymerase Chain Reaction (RT-PCR) and or IgM rapid test or Enzyme-linked Immunosorbent Assay (ELISA) as an **index** case within 48 hours of admission to Hasan Sadikin Hospital, Bandung, Indonesia. After confirmation of DENV infection in an admitted patient, a field visit was executed in the greater Bandung area to invite and enroll twenty individuals aged four years or older, living in or within a 100 m radius of the index case’s home as **community contact**. Pregnant women were eligible to participate. The exclusion criteria were a history of recent severe anemia, bleeding disorders, specific conditions including HIV infection, chronic hepatitis infection, or severe autoimmune diseases requiring immunosuppressant treatment. Upon informed consent approval, we recorded demographic information and clinical data and drew blood samples on the day of enrollment of all participants. Thereafter, a research nurse was assigned to monitor all enrolled community contacts for 14 days (Follow up days 1–14 (FD1-FD14)) for febrile episodes through daily temperature measurements and blood sample collection every 3–4 days.

If fever (≥ 37.5°C) was documented, blood specimens were drawn daily from the first day of fever onset (defined as day Zero (D0)) until two days after defervescent. Dengue confirmatory diagnostic and complete blood count were carried out in the initial (pre-febrile) and the acute blood samples from the febrile cases. A convalescent blood sample was obtained in all febrile participants two weeks after fever onset (D14). Dengue-confirmed patients were admitted to the hospital for observation if platelet counts fell below 150 x10^3^/μL or in case of other symptoms or signs of severe disease. If all dengue confirmatory diagnostic tests were negative, participants were asked to remain within the community cluster until the two-week follow-up period.

### Study case definitions, dengue diagnostics, and infection status

Dengue confirmatory diagnostics performed on blood samples collected from index and community contacts can be seen in **S2 Fig Index cases** were considered to have an acute DENV infection if they had symptoms or signs consistent with acute dengue together with a positive result of a dengue antibody IgM ELISA or IgM rapid test, or a positive result of a dengue RT-PCR within 48 hours after admission. **Community contact** samples were examined for antibody IgM rapid test and ELISA, RT-PCR, and/or virus isolation tests [8,9]. Blood samples gathered from all community contacts on enrolment day (FD1) were tested for IgM ELISA, dengue RT-PCR, and, later on, virus isolation. While the IgM ELISA was carried out to the last sample (FD14) to observe antibody seroconversion. For symptomatic cases, all subsequent samples were tested for dengue RT-PCR and, later on, virus isolation. Antibody seroconversion and type of immune response were determined in the paired acute (D0)-convalescent (D14) samples from symptomatic cases by dengue antibody IgM and IgG ELISA. For asymptomatic cases, paired first and last samples (FD1 and FD14) will be tested for IgG ELISA. The immune responses were classified into primary and secondary DENV infection; primary infection is defined if IgG anti-dengue is only detected in the convalescent sample and none detected in the acute sample; otherwise, it will be classified as a secondary DENV infection [10]. In symptomatic cases, dengue severity was classified as dengue fever (DF) or dengue hemorrhagic fever (DHF) using the 1997 WHO dengue criteria [11]. Based on the timing and result of the test, we further categorized the community contacts around the index cases into four groups: 1) non-dengue (ND) infection if no dengue confirmatory tests were positive in febrile cases, 2) Recent DENV infection (RD) if non-specific IgM antibody reactivity found without any evidence of an acute infection, 3) Enrollment dengue (ED); if an acute DENV infection was confirmed (positive RT-PCR/IgM seroconversion) in community contacts at the time of study enrollment, 4) Post-enrollment dengue (PED); if acute DENV infection is confirmed (positive RT-PCR/IgM seroconversion) within the 14 days follow-up period after study enrollment, with or without symptoms. Positive clusters were clusters where DENV infections were identified. An **asymptomatic** DENV infection was confirmed when there was no reported fever day or other illness and showed IgM seroconversion and/or positive RT-PCR or viral Isolation during two weeks of observation [10].

### Dengue serology tests

Commercial Panbio Dengue Duo Cassette, IgM, and IgG rapid test (Panbio, Brisbane, Australia) were used following the manufacturer’s instructions [12], the sensitivity of the IgM antibody rapid test is 77.8% (95%CI 75.5–80.1%) and the specificity is 90.6% (95%CI 88.9–92.3%) [13], while the sensitivity of the IgG rapid test range from 52% to 98% and specificity range from 58.3 to 100% [14,15]. IgM antibodies against dengue were measured using commercial ELISA kits Dengue Virus IgM DxSelect (Focus Technologies, Cypress, CA), following the manufacturer’s instructions. IgM index >1 and increased dengue IgM antibody index and seroconversion of dengue IgM antibody confirmed positive cases. The IgG ELISA was carried out using commercial kits Dengue Virus IgG DxSelect (Focus Technologies, Cypress, CA, USA) according to the manufacturer’s instructions. The sensitivity of IgM ELISA is 98.6% (95% CI 98.0–99.2%), and the specificity is 79.9% (95%CI 77.6–82.2%) [13], while the sensitivity of IgG ELISA is 96% (89.3–99.2%) and specificity 93% (89.7–96%) [16]. The index value was calculated by dividing the OD sample by the mean of calibrator ODs. Specimens with IgG index >1 were regarded as positive and IgG index <1 as negative [17]. The dengue Non-Structural-1 (NS1) rapid diagnostic tests were unavailable in Indonesia when the study was initiated.

### Dengue RT-PCR and viral isolation test

The RT-PCR assay was done following previously described methods [18] on all first blood samples (FD1) and acute sera to detect dengue virus RNA. Positive RT-PCR results were confirmed as a positive case. Virus isolation was performed in first samples (FD1), acutes, and PCR-positive samples following a previously described method [10]. In brief, virus isolation was performed in C6/36 tissue culture. Serum samples were separated into aliquots, diluted 1:10 in sterile tissue culture media, and applied to a confluent monolayer of C6/36 cells. After incubating at 25°C for one hour, the diluted serum was removed, and fresh tissue culture media was added. CPE (Cytopathic Effects) were observed after two passages of isolation, seven days per passage. Upon the CPE event or on day 14, cells were scraped from the plates and stained for the presence of the virus by standard immunofluorescence using flavivirus genus-reactive monoclonal antibody 4G2. Samples reactive against 4G2 were then subtyped by staining with DENV serotype-specific monoclonal antibodies.

### Dengue virus genotyping

The envelope genes from seven DENV-1 were sequenced as previously described [19]. In brief, viral RNA was extracted from virus isolates using the Qiamp Viral RNA mini kit (Qiagen, Germany). Three overlapping fragments covering the Envelope-NS1 genes (approximately 2700 bases) were amplified by RT-PCR using serotype-specific primer sets. Amplicons were purified, and the BigDye cycle sequencing kit (Applied Biosystems, USA) was used for sequencing reactions. Sequencing reactions were run on a 3130 XL Genetic Analyzer (Applied Biosystems), and sequence outputs were assembled using Sequencer software (Gene Codes, USA). Phylogenetic trees were generated using the Neighbor-Joining method with bootstrapping in MEGA 4 [20].

### Statistical analysis

Categorical data were presented as numbers and percentages, while numerical data as mean with standard deviation or median with interquartile range (IQR) or range. Comparison between groups was carried out using fisher’s exact test or chi^2^ for categorical data and Mann- Whitney u test for continuous data. Missing data were excluded from the analysis by running a complete case analysis. All statistical analyses were conveyed using STATA version 15.1 (Stata Corp, College Station, TX). The incidence per 1,000 person-years was 1,000 times the number of new dengue infections (PED) divided by the number of person-years in the new dataset (= 1,000*infections/ (sum(days)/ 365.25)) [21].

## Results

### Demographic characteristics of the index cases

From February 2005 –to April 2009, 97 hospitalized confirmed dengue cases were enrolled as index cases. The characteristics of index cases are shown in **Table 1**. Sixty-nine percent of the index cases were children. The overall median age was 12 years (IQR 7–21, range 4–46), and the median duration of fever at enrollment was five days (range 1–12). Among these index cases, 23 (24%) subjects were positive for RT-PCR and IgM ELISA, and eight (8%) subjects were positive for RT-PCR only. The remaining 66 (68%) were positive for IgM only (**S1 Table**). Most index cases were classified as severe dengue; Dengue Hemorrhagic Fever (DHF) (n = 47, 48.5%), and Dengue Shock Syndrome (DSS) (n = 11, 11.3%). We saw a trend that primary dengue was more frequent in Dengue Fever (DF) cases (13.5%) vs. 4.3% in DHF cases. Secondary dengue was found most in DSS cases (100%), in DHF (93.6%), and the least in DF (82%) cases (**Table 1**). All four dengue serotypes were discovered, DENV-3 being the most common (15/31 cases, 48.4%).

**Table 1:** Characteristics of the dengue index cases.

Characteristics	DF (n = 39)	DHF (n = 47)	DSS (n = 11)	p-value
Female, (n, %)	10 (25.6)	21 (44.7)	4 (36.4)	0.19^1^
Age (year) (n,%)				
- Children (<18)	25 (64.0)	31 (66.0)	11 (100.0)	0.04^1^
- Adult (≥18)	14 (36.0)	16 (34.0)	-	
Age (year) (Median, IQR)	13 (9–26)	13 (7–21)	9 (6–11)	0.04^2^
Fever duration (Day) (Median, range)	6(1–8)	5 (1–9)	5 (1–12)	0.93^2^
RT-PCR Result (n,%)				0.96^1^
- DENV-1	2 (5.1)	4 (8.5)	-	
- DENV-2	4 (10.3)	3 (6.4)	-	
- DENV-3	7 (17.9)	6 (12.8)	2 (18.2)	
- DENV-4	1 (2.6)	2 (4.3)	-	
- Negative	25 (64.1)	32 (68.1)	9 (81.8)	
Anti-dengue IgM Result (n,%)				0.48^1^
- Positive	34 (87.2)	44 (93.6)	11 (100.0)	
- Negative	5 (12.8)	3 (6.4)	-	
Antibody response (n,%)	N = 37	N = 46		0.26^1^
- Primary	5 (13.5)	2 (4.3)	-	
- Secondary	33 (86.8)	44 (95.6)	11 (100.0)	

^1^Fisher’s exact test

^2^Kruskal-Wallis rank test

### Dengue incidence in the community contacts clusters

**Fig 1:**
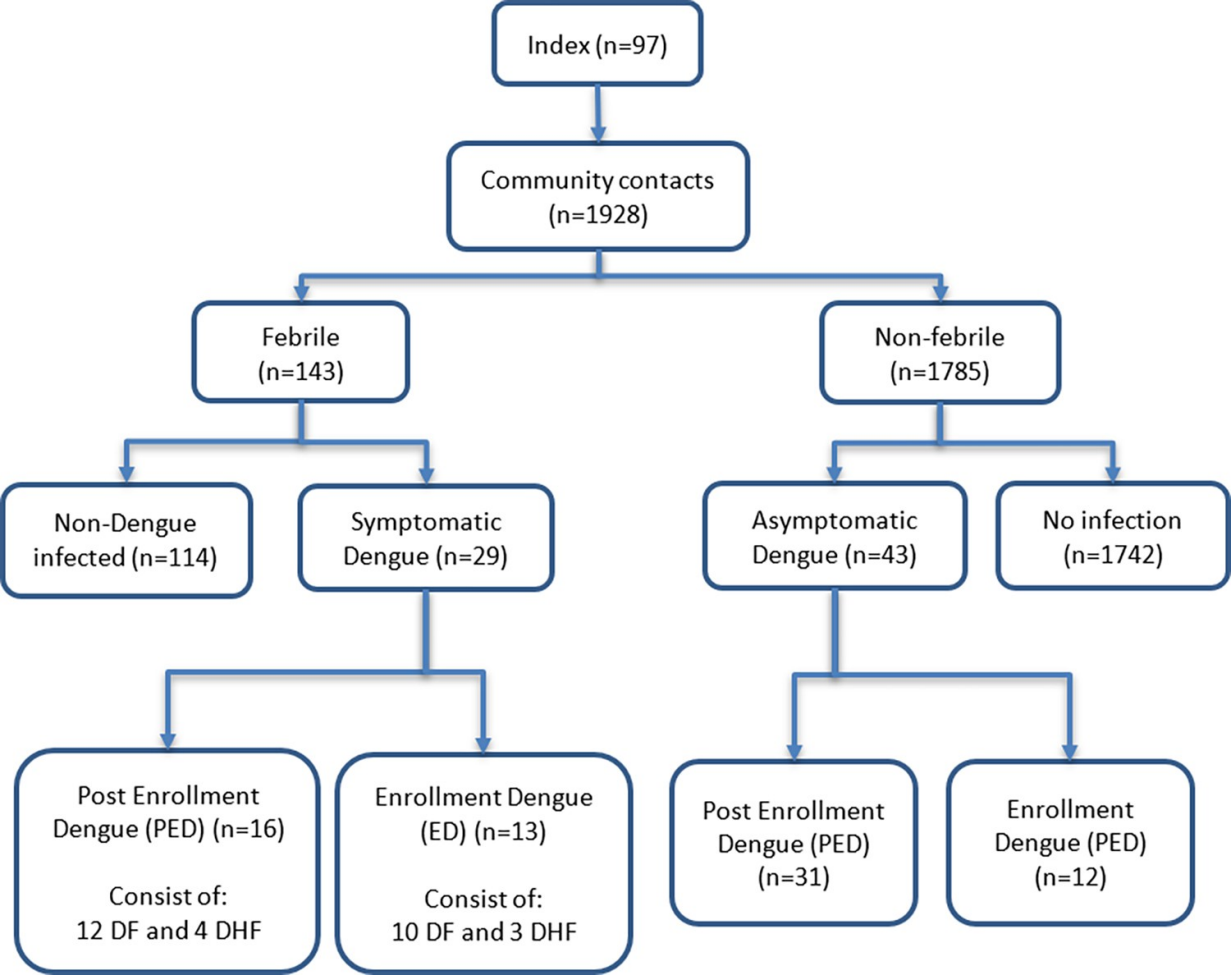
Study flow chart. **Post-Enrollment Dengue (PED)** = dengue fever is confirmed after enrollment. **Enrollment Dengue (ED)** = dengue fever is diagnosed on the day of enrollment (based on positive RT-PCR and or seroconversion of IgM ELISA accompanied by thrombocytopenia). DF: Dengue Fever, DHF: Dengue Hemorrhagic Fever.

A total of 97 clusters were established with 1928 community contacts, consisting of 231 family members and 1697 neighbors. Among the contacts, 722 were children, and 1206 were adults. The median age is 28 years (IQR: 10–43 years); 70.7% were female. A median of 19 (min. 16 max. 20) subjects per cluster was followed up for two weeks. Overall, an acute DENV infection was diagnosed in 72 (3.7%) community contacts, of whom 25 had the infection at enrolment (‘enrolment dengue’; ED) and 47 during the follow-up (‘post-enrolment dengue’; PED) (**Fig 1**). The 72 contacts belonged to 46 positive clusters; five of the clusters were situated around an index case that was RT-PCR positive only (5/8, or 63%), and nine clusters were around an index case with both positive RT-PCR and IgM (9/23, or 39%), and 32 clusters were living around an index case which was only IgM positive (32/66, or 39%) (**S1 Table**). In 3% of the community clusters, we did not find recent DENV infections or ED and PED cases in their community contacts, suggesting that the index cases in these clusters might be infected outside their community. Among the community contacts of these negative clusters, five community participants were previously reported to be infected by chikungunya [22]. The diversity of dengue serotypes between index and their community contacts within the same cluster is presented in the **S2 Table**.

Among the 1856 cases without acute dengue, 256 (13.7%) had a positive dengue IgM antibody without any other indication of acute DENV infection (recent dengue). Out of 72 confirmed dengue cases, 42 (58.3%) were below 18 years old, and 50 (69%) had positive RT-PCR (20 adults and 30 children). The predominant infecting serotypes were DENV-3 (n = 21; 42%), followed by DENV-2 (n = 13; 26%), DENV-1 (n = 11; 22%) and DENV-4 (n = 5; 10%). Primary DENV infection was detected in 18 cases (5 adults and 13 children, or 25%), and secondary DENV infection in 54 cases (25 adults and 29 children, or 75%) (**Table 2**).

**Table 2:** Description of dengue infected community in the cluster, stratified by post-enrollment (PED) or at-enrollment (ED) time.

Characteristics and test results	PED Cases (n = 47)		ED Cases (n = 25)		p-value
	Symptomatic (n = 16)	Asymptomatic (n = 31)	Symptomatic (n = 13)	Asymptomatic (n = 12)	
Female (n,%)	10 (62.5)	21 (67.7)	5 (38.5)	7 (58.3)	0.34^1^
Age (year) (n,%)					0.09^1^
- Children (<18)	11 (68.7)	13 (41.9)	10 (76.9)	8 (66.7)	
- Adult (≥18)	5 (31.3)	18 (58.1)	3 (23.1)	4 (33.3)	
Age (year) (Median, IQR)	10 (8–10)	21 (9–21)	8 (7–8)	13 (7–13)	0.06^2^
Viremia duration (Day) (Median, IQR)					
- Primary	6 (5–6)		3 (0–3)		
- Secondary	5 (3–5)		1.5 (1–1.5)		
Dengue RT-PCR					<0.001^1^
- DENV-1	6 (37.5)	1 (3.2)	3 (23.1)	1 (8.3)	
- DENV-2	2 (12.5)	7 (22.6)	2 (15.4)	2 (16.7)	
- DENV-3	7 (43.7)	1 (3.2)	7 (53.8)	6 (50)	
- DENV-4	1 (6.3)	3 (9.7)	0	1 (8.3)	
- Negative	0	19 (61.3)	1 (7.7)	2 (16.7)	
Clinical Classification and Antibody Response					
- Primary (n,%)	5 (100.0)	7 (100.0)	3 (100.0)	3 (100.0)	0.96^1^
- DF	4 (80.0)	NA	3 (100.0)	NA	
- DHF	1 (20.0)	NA	0 (0.0)	NA	
- Secondary (n,%)	11 (100.0)	24 (100.0)	10 (100.0)	9 (100.0)	
- DF	8 (72.7)	NA	7 (70.0)	NA	
- DHF	3 (27.3)	NA	3 (30.0)	NA	

Viremia duration (Day) = the last positive RT-PCR date- first positive RT-PCR result date. NA = Not Applicable.

^1^Fisher’s exact test

^2^Mann-Whitney test

The highest number of PED cases was in the year 2009 (8 cases within four months), followed by the year 2007 and 2008 (13 cases within 12 months), 2005 (8 cases/12 months), 2006 (5 cases/12 months) (**S3 Table**). The calculated incidence rate of dengue infection (PED) was 636 cases per 1,000 person-years (95% Confidence interval (CI) 588 to 687 cases per 1,000 person-years) of follow-up (**S3 Table**). The incidence rate of symptomatic cases was 217 cases per 1,000 person-years (95% CI 189 to 248 cases per 1,000 person-years) of follow-up. The incidence rate of asymptomatic cases was 419 cases per 1,000 person-years (95% CI 380 to 461 per 1,000 person-years) of follow-up.

### Symptomatic and asymptomatic dengue among community contacts

Among the 72 acute dengue cases, 29 (40%) had clinical symptoms (8 adults and 21 children), and 43 (60%; 22 adults and 21 children) remained asymptomatic (2.2% from all community contacts). Among the symptomatic cases, 16 (of whom 11 children) developed dengue during follow-up (PED), whereas 13 (10 were children) already had acute dengue at enrolment (ED). In addition, DENV-3 was the predominant infecting serotype (n = 14) among symptomatic participants, and secondary DENV infection was identified in 21 (72.4%) subjects. Asymptomatic cases consisted of 31 PED (13 were children) and 12 ED (8 were children); DEN-2 was predominant (n = 9), and secondary DENV infection was identified in 33 (76.7%) subjects (**Table 3**). The overall asymptomatic-to-symptomatic ratio was 1.5:1, and the asymptomatic-to-symptomatic ratio in children was 1:1. Children in our study had higher OR (2.75 OR, 95% CI: 1.02–7.42) than adults for having symptomatic dengue cases.

**Table 3:** Characteristics of dengue symptomatic and asymptomatic cases in the community.

Characteristics and test results	Symptomatic (n = 29)	Asymptomatic (n = 43)	p-value
Female (n,%)	15 (51.7)	28 (65.1)	0.25^**3**^
Age (year) (n,%)			**0.04** ^**3**^
- Children (<18)	21 (72.4)	21 (48.8)	
- Adult (≥18)	8 (27.6)	22 (51.2)	
Age (year) (Median, IQR)	10 (7–19)	19 (8–45)	**0.02** ^**2**^
Viremia duration (Day) (Median, IQR)			
- Primary	5.5 (2.0–7)		
- Secondary	3.0 (2.0–5)		
Dengue RT-PCR			**<0.001** ^**1**^
- DENV-1	9 (31.0)	2 (4.7)	
- DENV-2	4 (13.8)	9 (20.9)	
- DENV-3	14 (48.3)	7 (16.3)	
- DENV-4	1 (3.4)	4 (9.3)	
- Negative	1 (3.4)	21 (48.8)	
Clinical Classification and Antibody Response			0.67^1^*
- Primary	8 (27.6)	10 (23.3)	
- DF	7 (87.5)		
- DHF	1 (12.5)		
- Secondary	21 (72.4)	33 (76.7)	
- DF	15 (71.4)		
- DHF	6 (28.6)		

Viremia duration (Day) = the last positive RT-PCR date- first positive RT-PCR result date.

^1^Fisher’s exact test

^2^Mann-Whitney test

^3^Chi-square

*comparing primary and secondary infection among symptomatic group

### Viremia in symptomatic dengue cases

Viremia was detected in 28 of the 29 symptomatic dengue cases (97%); 16 were PED, and 12 were ED (**Table 4**). These cases were followed up and assessed daily for RT-PCR and IgM ELISA. Pre-febrile viremia was detected in 8 cases, with the earliest viremia identified seven days before the onset of fever (one case), followed by two days before the onset of fever (three cases), and one day before the onset of fever (four cases). On average, the duration of viremia in PED cases was 4.9 days (range 1–13 days). We could not calculate the duration of viremia in ED cases because we did not have information regarding the starting date of fever and viremia. Our study also documented viremia in asymptomatic cases (**Table 5)**; however, we could not determine the length of viremia in asymptomatic cases since no daily sample testing data was available.

**Table 4:** Viremia in symptomatic dengue cases.

ID	Age	Sex	Clinical Manifestation	Dengue Serotype	Immune Response	Days of fever																										
						D-14	D-11	D-10	D-9	D-8	D-7	D-6	D-5	D-4	D-3	D-2	D-1	D0	D1	D2	D3	D4	D5	D6	D7	D8	D9	D10	D11	D12	D13	D14
PED1	20	1	DF	DENV-3	1°	○ *					● *			● *				● *	● #	● #	● #	○ #	● *									
PED2	10	1	DF	DENV-3	2°							○ *			○ *			● *			● *	● *	● *	● *								
PED3	38	2	DF	DENV-3	2°					○ *			*			● *	● *	● *			● ● #											
PED4	15	1	DHF II	DENV-1	2°					○ *			○ *			● *		● *	● *	● *	● *			○ #	○ #							
PED5	10	2	DF	DENV-3	1°			○ *								● *		● *	● *	● ● *	● #	○ #										
PED6	9	1	DHF I	DENV-3	2°					○ *				○ *			● *	● *			● *	● *	○ #	○ #			#					
PED7	6	2	DF	DENV-1	1°		○ *						○ *				● *	● *	● *	● *	● *	○ #	○ #		○ #							
PED8	7	2	DHF I	DENV-3	2°									○ *			● *	● *	*	○ *	● *	● *		○ *			#					
PED9	23	2	DF	DENV-1	2°									○ *			● *	● *		● *	● *	○ *	○ #	#	#		○ #					
PED10	4	1	DF	DENV-2	2°										○ *			● *	● *	● *	● #	● #	○ #	○	○							
PED11	10	2	DF	DENV-4	2°			○ *				○ *			○ *			● *		● *	○ *											
PED12	5	2	DHF II	DENV-1	2°			○ *			*			○ *				● *	● *	● *	○ #	○ #										
PED13	35	2	DF	DENV-3	2°			○ *							○ *			● *	● *	● *	○ *	○ *										
PED14	30	2	DF	DENV-1	2°			○ *			*			○ *				● *	● *													
PED15	10	2	DF	DENV-2	2°				○ *									● #	● #			○ #										
PED16	10	1	DF	DENV-1	1°												○ *	● *			○ *		*							○ *		
ED1	33	1	DF	DENV-2	1°													● *	● *	● *	● #	● #	● #	○ #	● #	○ #						
ED2	8	1	DHF II	DENV-3	2°													● #	○ #	○ #	○ #		● #	○ #			○ #			○ #		
ED3	6	1	DHF I	DENV-3	2°													● *		● *	● *	○ #	○ #	○ #	○ #	○ #					○ #	
ED4	6	1	DF	DENV-3	1°													● *	● *	● *	○ *	○ *		○ #								
ED5	12	2	DF	DENV-1	2°													● *	● *			○ #			#			#				#
ED6	11	2	DF	DENV-1	2°													● *	● *			○ #			#			#				○ #
ED7	8	1	DHF II	DENV-3	2°													● *	● *	○ *	○ *	○ #	#	○ #	#	○ #						
ED8	7	2	DF	DENV-1	2°													● *	○ #			○ #			#			#				○ #
ED9	30	1	DF	DENV-3	2°													● *			○ *						○ #			○ #		
ED10	9	1	DF	DENV-2	2°													● *														
ED11	19	2	DF	DENV-3	2°													● *			○ #		○ #									
ED12	8	2	DF	DENV-3	2°													● *	○ #													

Reverse Transcriptase PCR (RT-PCR) detected viremia in 28 symptomatic dengue cases found in the community contacts, consisting of 16 Post Enrollment Dengue (PED1-PED16) and 12 Enrollment Dengue (ED1-ED12) cases. For ED cases, enrollment day is considered as the first day of fever.

Notes: Day 0 = Onset of fever; Immune Response: 1° = Primary Infection, 2° = Secondary Infection; Sex: 1 = Male, 2 = Female.

● = Positive RT-PCR

○ = Negative RT-PCR

# = Positive IgM ELISA

* = Negative IgM ELISA

**Table 5:** Viremia in asymptomatic dengue cases.

ID	Age	Sex	Dengue Serotype	Immune Response	Follow up day																		
					FD1	FD2	FD3	FD4	FD5	FD6	FD7	FD8	FD9	FD10	FD11	FD12	FD13	FD14	FD15	FD16	FD17	FD18	FD19
ASED1	11	1	DENV-1	2°	● *				○ #														
ASED3	8	1	DENV-3	1°	● *																		
ASED4	15	2	DENV-3	2°	● #			#										○ #					
ASED6	25	1	DENV-4	2°	● *			● #			○ #			#					○ #				
ASED7	17	2	DENV-3	2°	●			●				○ #			○ #								
ASED8	6	2	DENV-3	2°	● #																		
ASED9	4	1	DENV-3	1°	● #																		
ASED10	70	2	DENV-3	2°	● *				○ #	○ #	#												
ASED11	33	2	DENV-2	1°	● *			○ *		○ *								○ *					
ASED12	45	1	DENV-2	2°	● *							○ #						○ #					
ASPED2	9	2	DENV-4	2°	○ *				● *						○ #			○ #					
ASPED4	20	2	DENV-2	1°	○ *			● *		● *	● *	● *		● #	● #	● #	● #	○ #					
ASPED11	6	1	DENV-2	2°	○ *			○ *			○ *				○ *			● *					
ASPED12	44	2	DENV-4	2°	○ *				● #			○ #				#			○ #				
ASPED15	32	2	DENV-2	2°	○ *							●							○ *				
ASPED16	55	2	DENV-3	2°	○ *			○				●			*				○ *				
ASPED19	36	2	DENV-4	2°	○ *													●					
ASPED20	19	2	DENV-2	2°	○ *			● *										○ *					
ASPED23	29	2	DENV-2	2°	○ *				● *			○ #			○ #			○ #					
ASPED24	5	1	DENV-1	1°	○ *						● #							○ *					
ASPED26	9	2	DENV-2	1°	○ *										● *								
ASPED28	47	2	DENV-2	1°	○ *										○ *			● *		● *	● *	● *	● #

Reverse Transcriptase PCR (RT-PCR) detected viremia in 22 asymptomatic dengue cases found in the community contacts, consisting of 12 Post Enrollment Dengue (ASPED2-ASPED28) and 10 Enrollment Dengue (ASED1-ASED12) cases.

Notes: FD1 = Enrollment Day; Immune Response: 1° = Primary Infection, 2° = Secondary Infection; Sex: 1 = Male, 2 = Female.

● = Positive RT-PCR

○ = Negative RT-PCR

# = Positive IgM ELISA

* = Negative IgM ELISA

## Discussion

Using a community cluster study design, we documented viremia in eight out of sixteen symptomatic post-enrollment dengue cases during the pre-illness period. The longest period of viremia in pre-illness lasted for seven days. Furthermore, we found 1.5 asymptomatic DENV infections for each symptomatic case. With the active surveillance embedded in the study design, we calculated the incidence of DENV infection in the year 2005–2009 to be 636 cases per 1,000 person-years of follow-up.

Viremia is an important indicator of the level of dengue transmission in the community. A previous cluster study reported that DENV-infected humans can transmit the virus to mosquitoes from 2 days before until two to six days after the development of fever [5,23]. Infected mosquitoes could transmit the virus up to 11 days after the extrinsic incubation period [23]. Here we show that viremia can already be detected up to seven days before and after fever onset, even though this was only found in one participant. This phenomenon was not found in previous community cluster studies that reported presymptomatic viremia [4,5], as only one study reported kinetics of dengue viremia detected three days before the fever developed [5]. Previously, our group also reported presymptomatic chikungunya viremia, which lasted as early as six days prior development of fever within the community contacts [22]. Our study design acquired presymptomatic samples and observed the complete length of viremia from arboviral diseases. Here, we urge comprehensive and sustainable vector control interventions to cut the transmission of arboviral infections.

Asymptomatically infected subjects may spread DENV but remain unnoticed [5]. We found 1.5 asymptomatic individuals for each symptomatic individual, and almost half of them were children. DENV-2 was the most common serotype among asymptomatic participants. Overall, 2.2% of the community contacts had an asymptomatic infection, lower than the 7.4% previously reported from Thailand in 2012–2015 [4]. Our ratio of asymptomatic-to-symptomatic cases (1.5:1) was lower than a previous report from West Java (2.6:1) [10] and lower than in children in Thailand (1.8:1) [24]. Symptomatic cases in our study were more frequent in young subjects and those carrying DENV-3. These findings confirmed other studies which found similar factors related to symptomatic and more severe dengue [4,25].

The high incidence rate calculated in this study was similar to a previous report from a cluster study in Jakarta [8]. Our calculated incidence rate is five times and 37 times higher than those previously reported in cohort studies from the great Bandung area around the same study periods [9,10]. This wide difference may be attributed to the design of our study, which allowed the selection of active surveillance areas in high-risk populations where the communities are known to have recent or current circulating DENV infections.

The index cases in our study were mostly enrolled in the late acute phase of infection as the median duration of fever was five days, and dengue IgM antibody could already be detected. Ideally, enrollment would have occurred at an earlier phase of infection as these subjects may have been more infectious. A previous study showed that human-to-mosquito transmission decreases with increasing illness days and the presence of IgM and IgG titers [5]. Therefore, antibody and antigen or RT-PCR testing are recommended to detect early DENV transmission in the community. Furthermore, using IgM antibody serology alone has more limitations, as IgM antibody titers do not rise immediately after infection, and in 20% of secondary DENV infections, IgM antibody titers might be low and result in undetectable levels [26].

The sensitivity of the IgM RDT assay used in this study is not very high (77.8%); thus, our study might miss cases when viremia was negative and antibodies had already appeared. To minimize missing cases, we used IgM ELISA with very high sensitivity (almost 100%), but we could not eliminate the possibility of missing some DENV infection cases. However, it meant that the real number of dengue cases should be higher than we reported. So far, other flaviviruses, such as Japanese encephalitis, West Nile, and Zika virus, have been reported in Indonesia [27–29]. The exposure to other flaviviruses before our study could cause our subjects to have pre-existing antibodies. However, we did not test for other flaviviruses to rule out the cross-reaction due to other flaviviruses. In general, the overall number of dengue cases found using a similar design to our study could be different due to assay performance. But we could not estimate whether the number would be higher or lower.

Our study is suited to determine the distribution of DENV serotypes, previous dengue immune status, other risk factors, and clinical outcomes. Although the results of the statistical tests were insignificant and our findings are limited due to the low number of individuals in some subgroups, our observations showed that DHF and DSS are caused mainly by secondary dengue infections, and there were more DENV-3 than DENV-2 in DHF and DSS cases. DENV-3 is the predominant serotype in our study area during the study period [10].

In our experience, a community cluster study proved to be an efficient and practical way to study acute dengue infections and measure their outcomes. The cluster study design allows monitoring individuals before the onset of illness in the setting of a high probability of DENV transmission in and around a home. We calculated the incidence rate for 1000 person-years despite the fact that our observation period only lasted two weeks in an area with a very high-risk population: our finding, therefore, did not reflect the overall transmission in other areas, nor was it adequately precise enough to estimate the natural national incidence rate. Furthermore, our high case rate should be interpreted cautiously due to selection bias where we enrolled subjects living nearby index cases. Hence, our study case rate was only comparable to a similar report from other high-risk populations and should not be compared to the general population or other studies with lower-risk populations.

Although our study was conducted almost two decades ago, the findings of dengue incidence and symptomatic and asymptomatic cases are still relevant compared to current reports. Our study design allows us to observe the length of prefebrile viremia and asymptomatic viremia cases, an important dengue transmission reservoir.

## Conclusion

The high number of asymptomatic infections and the significance of a longer duration of unnoticed virus transmission, as our data indicate, could lead to prolonging the presence of a potential reservoir for the transmission of DENV infections, thus supporting the need for comprehensive and sustainable vector control interventions.

## Supporting information

S1 Checklist STROBE checklist. (DOC)

S1 Fig Cluster study design. (TIF)

S2 Fig Laboratory testing for index and community contacts. (TIF)

S1 Table Characteristics of index cases, comparing those with and without confirmed dengue in the community contacts. (DOCX)

S2 Table The diversity of dengue serotypes between index and their community contacts within the same cluster. (DOCX)

S3 Table Dengue serotype distribution from 2005 to 2009 and incidence rate per year. (DOCX)

S1 Data Appendix 1. (XLSX)
