# Supplementary figures and images for: Dengue incidence and length of viremia by RT-PCR in a prospective observational community contact cluster study from 2005–2009 in Indonesia
Source: PLoS Negl Trop Dis. 2023 Feb 6;17(2):e0011104. doi: 10.1371/journal.pntd.0011104 (PMC9901748; doi:10.1371/journal.pntd.0011104)

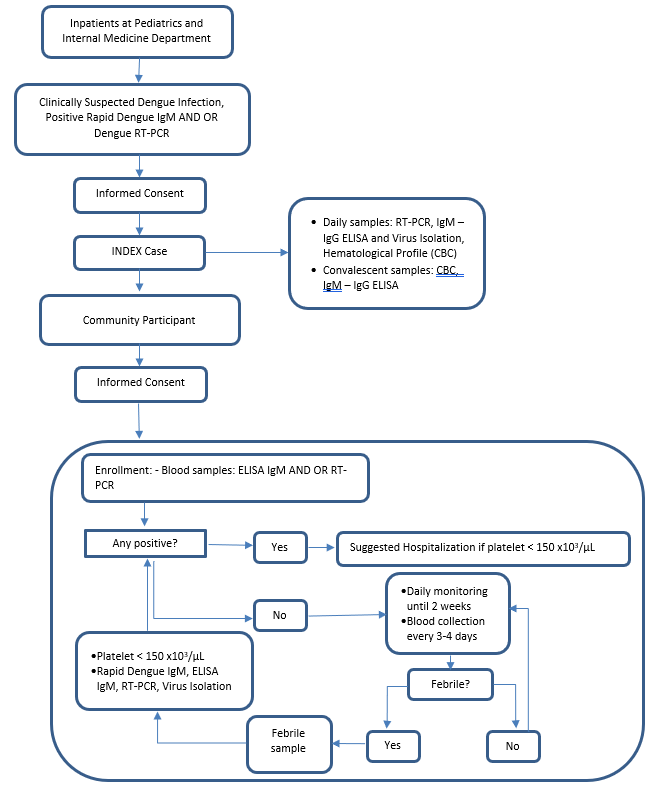

Supplement: S1 Fig — (TIF) [file pntd.0011104.s002.tif]

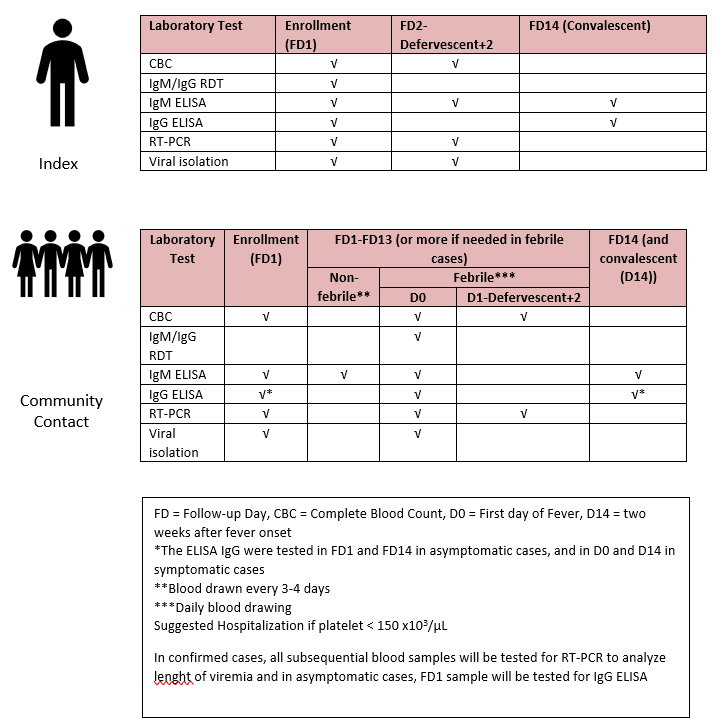

Supplement: S2 Fig — (TIF) [file pntd.0011104.s003.tif]
