# Supplementary material for: Dengue incidence and length of viremia by RT-PCR in a prospective observational community contact cluster study from 2005–2009 in Indonesia
Source: PLoS Negl Trop Dis. 2023 Feb 6;17(2):e0011104. doi: 10.1371/journal.pntd.0011104 (PMC9901748; doi:10.1371/journal.pntd.0011104)
Supplement: S1 Table — (DOCX) [file pntd.0011104.s004.docx]

S1 Table. Characteristics of Index cases, comparing those with and without confirmed dengue in the community contacts

| **Characteristics** | **Index (n=97)** | | | |
| --- | --- | --- | --- | --- |
|  | **Index with cases in Community Contacts (n=46)** | **Index without cases in Community Contacts (n=51)** | **p-value** |  |
| Laboratory confirmation (n, %) |  |  | 0.51^1^ |  |
| - RT-PCR (+), IgM (-) | 5 (10.9)^a^ | 3 (5.9) |  |  |
| - RT-PCR (+), IgM (+) | 9 (19.6)^b^ | 14 (27.5) |  |  |
| - RT-PCR (-), IgM (+) | 32 (69.5)^c^ | 34 (66.7) |  |  |
| Fever duration (Day) (Median, range) | 5 (1-8) | 6(1-12) | 0.28^2^ |  |
| Fever duration (Day) (Median, range) |  |  |  |  |
| - RT-PCR (+), IgM (-) | 4(3-7) | 4 (4-6) | 0.42^2^ |  |
| - RT-PCR (+), IgM (+) | 5(1-8) | 5.5 (1-9) | 0.58^2^ |  |
| - RT-PCR (-), IgM (+) | 5.5 (1-8) | 6 (1-12) | 0.53^2^ |  |

The PED and ED cases found in the community; a=7 PED and 2 ED, b=10 PED and 5 ED, c=30 PED and 18 ED. ^1^Fisher’s exact test ^2^Mann-Whitney test.
