# Supplementary material for: Dengue incidence and length of viremia by RT-PCR in a prospective observational community contact cluster study from 2005–2009 in Indonesia
Source: PLoS Negl Trop Dis. 2023 Feb 6;17(2):e0011104. doi: 10.1371/journal.pntd.0011104 (PMC9901748; doi:10.1371/journal.pntd.0011104)
Supplement: S2 Table — (DOCX) [file pntd.0011104.s005.docx]

**S2 Table.** The diversity of dengue serotypes between Index and their community contacts within the same cluster

| **Year** | **Cluster No.** | **Dengue Serotype in Index Case** | **Total Dengue Community Contact Cases** | **Community Contact Case (ID/Type Infection/Dengue RT-PCR Result/Immune response)** |
| --- | --- | --- | --- | --- |
| 2005 | 7 | DEN-3 | 1 | ASED4/Asymptomatic ED/DEN-3/Secondary |
| 2005 | 14 | DEN-3 | 4 | ED11/Symptomatic ED/DEN-3/Secondary |
|  |  |  |  | ED3/Symptomatic ED/DEN-3/Secondary |
|  |  |  |  | ASPED3/Asymptomatic PED/NEG/Secondary |
|  |  |  |  | ED7/Symptomatic ED/DEN-3/Secondary |
| 2007 | 39 | DEN-2 | 1 | ASPED8/Asymptomatic PED/NEG/Secondary |
| 2007 | 53 | DEN-4 | 2 | ASPED12/Asymptomatic PED/DEN-4/Secondary |
|  |  |  |  | ASPED13/Asymptomatic PED/NEG/Secondary |
| 2007 | 56 | DEN-1 | 1 | ASPED14/Asymptomatic PED/NEG/Primary |
| 2007 | 57 | DEN-3 | 2 | ASPED15/Asymptomatic PED/DEN-2/Secondary |
|  |  |  |  | ASPED16/Asymptomatic PED/DEN-3/Secondary |
| 2008 | 60 | DEN-4 | 1 | ASPED18/Asymptomatic PED/NEG/Secondary |
| 2008 | 65 | DEN-3 | 1 | ASPED20/Asymptomatic PED/DEN-2/Secondary |
| 2008 | 81 | DEN-1 | 2 | ASED9/Asymptomatic ED/DEN-3/Primary |
|  |  |  |  | PED14/Symptomatic PED/DEN-1/Secondary |
| 2008 | 83 | DEN-3 | 2 | PED13/Symptomatic PED/DEN-3/Secondary |
|  |  |  |  | ASED10/Asymptomatic ED/DEN-3/Secondary |
| 2008 | 87 | DEN-1 | 1 | ASPED25/Asymptomatic PED/NEG/Secondary |
| 2009 | 93 | DEN-3 | 1 | ASPED27/Asymptomatic PED/NEG/Secondary |
| 2009 | 94 | DEN-3 | 4 | ASED2/Asymptomatic ED/DEN-2/Secondary |
|  |  |  |  | ASPED28/Asymptomatic PED/DEN-2/Primary |
|  |  |  |  | ASPED29/Asymptomatic PED/NEG/Secondary |
|  |  |  |  | ASPED30/Asymptomatic PED/NEG/Secondary |
| 2009 | 99 | DEN-1 | 1 | ASPED31/Asymptomatic PED/NEG/Secondary |
