# Supplementary material for: Dengue incidence and length of viremia by RT-PCR in a prospective observational community contact cluster study from 2005–2009 in Indonesia
Source: PLoS Negl Trop Dis. 2023 Feb 6;17(2):e0011104. doi: 10.1371/journal.pntd.0011104 (PMC9901748; doi:10.1371/journal.pntd.0011104)
Supplement: S3 Table — (DOCX) [file pntd.0011104.s006.docx]

S3 Table. Dengue serotype distribution from 2005 to 2009 and incidence rate per year.

| **Year** | **Group** | **Total** | **Dengue Serotype** | | | | **Incidence rate (/1000 person years) (95% CI)** |
| --- | --- | --- | --- | --- | --- | --- | --- |
|  |  |  | **DEN-1** | **DEN-2** | **DEN-3** | **DEN-4** |  |
| 2005 | Index | 21 | 0 | 3 | 2 | 0 |  |
|  | PED | 8 | 2 | 0 | 2 | 2 | 108 (89 to 130) |
|  | ED | 14 | 4 | 1 | 7 | 0 |  |
| 2006 | Index | 15 | 0 | 0 | 1 | 0 |  |
|  | PED | 5 | 0 | 1 | 2 | 0 | 68 (53 to 86) |
|  | ED | 2 | 0 | 0 | 2 | 0 |  |
| 2007 | Index | 24 | 1 | 2 | 5 | 2 |  |
|  | PED | 13 | 0 | 3 | 2 | 1 | 176 (151 to 204) |
|  | ED | 3 | 0 | 1 | 1 | 1 |  |
| 2008 | Index | 26 | 3 | 1 | 5 | 1 |  |
|  | PED | 13 | 4 | 2 | 2 | 1 | 176 (151 to 204) |
|  | ED | 5 | 0 | 1 | 3 | 0 |  |
| 2009 | Index | 11 | 2 | 1 | 2 | 0 |  |
|  | PED | 8 | 1 | 3 | 0 | 0 | 108 (89 to 130) |
|  | ED | 1 | 0 | 1 | 0 | 0 |  |
|  |  |  |  |  |  | **TOTAL** | **636 (588 to 687)** |
